# Symptoms—A Study

**Published:** 1897-03

**Authors:** Stuart Close

**Affiliations:** Brooklyn, N. Y.


### SYMPTOMS—A STUDY.

STUART CLOSE, M. D., BROOKLYN, N. Y.

Read before the Brooklyn Hahnemannian Union.

The term symptom is like charity, in that it "covers a multitude of sins." One of the first realizations that dawns upon the mind of the student of Homœopathy is the ubiquity and importance of the symptom. He is introduced into what seems a perfect wilderness of symptoms, the density and extent of which is both confusing and discouraging. After a little study of the situation, however, openings appear. Entering, he finds the openings becoming paths, tortuous at first, but soon becoming wider, straighter, and more orderly, until at last he enters upon broad and sunny highways, with beautiful vistas, along which he may travel pleasantly in pursuit of his ideal.

That ideal should be, and with the true physician is, the speedy, gentle, and permanent restoration to health of those who are sick. It can be attained only by fulfilling the requirements of the law of cure; by entering into and working harmoniously with the divine plan for the redemption of the race from the bondage of sickness, sin, and death.

The remedial law, the harmonizing principle, in which the Divine Will is expressed, was phrased by Hahnemann in the formula, *similia similibus curantur*. Just to the extent we grasp and apply that principle on the various planes of man's being do we bring him into harmony with the divine order of things and minister to his welfare.

Homœopathy is a purely inductive science. It deals at first hand with facts, in accordance with a well-established and universal principle. Its foundations are laid in a mass of facts and observations so vast as to be simply amazing, but this mass has been analyzed, and its component parts so classified as to be readily available for the purposes of our art. In this art no fact is too insignificant to be overlooked, no true observation of phenomena too occult to be ignored. There is a place and use for all. The symptom which appears trifling and insignificant to the uninformed or superficial observer, in the hands of the master may assume the highest importance and become the key which opens up a difficult case.

The preliminary study and experience we have all had. We are daily using the knowledge we have attained, and verifying the truth of our principles. But it is profitable for us occasionally to review our lessons; to gather up the facts we have gained, the conceptions we have formed, and hold them anew before the mind for contemplation. Therefore it has seemed to me well to review a little of what we know about symptoms; their nature, varieties, relative value and uses.

Upon our view of this subject depends more than appears at first sight. It is really fundamental. Our conception of what a symptom is governs very largely our whole practical work as homœopathic physicians.

A true conception of a symptom is not only a guide to the examination of a case and the selection of the remedy, but a standard by which to measure the value of a proving, a clinical case, an examination record, or the professions of a new-coming *confrère*. Ignorance of the true nature and definition of a symptom on the part of provers, and directors of provings, has given us many records which are worthless, and filled our journals with reports of cases which served no valuable purpose except to float their authors' names on the sea of printer's ink.

Many of our modern provings are flagrant examples of the truth of these assertions. The prover had a headache. He felt very tired. He was restless at night and couldn't sleep. He felt as if he was going to have a fever, and carefully recorded his temperature. His head was muddled but he resolutely rose to the occasion and fulfilled the requirements of modern science by making an elaborate qualitative and quantitative analysis of his urine, by which we learn that there was a slight increase in the amount of earthy phosphate and a perceptible decrease in urophæin. Lest we should not be sufficiently impressed with his scientific attainments and devotion to the cause of homœopathic provings, he adds extensive sphygmographic tracings of his pulse! But he utterly fails to give us any simple, intelligible, clearly defined statement of his actual symptoms or the condition under which they appeared. That is a matter in which he has not been instructed. Possibly he has been falsely instructed and regards such trifles as beneath his notice.

Let us see what our leading thinkers have said on the subject of symptoms. Hahnemann defines symptoms as "any manifestation of a deviation from a former state of health, perceptible by the patient himself, the individuals around him, or the physician." Again as "the expression of disease in the sensations and functions of that side of the organism exposed to the senses of the physician and bystanders." Also as "evidences of the operation of influences which disturb the harmonious play of the functions, the vital principle as a spiritual dynamis." Symptoms, then, are the language of nature by which the organism expresses its sufferings. Symptoms are the elements with which the physician has to deal, the basis of all his practical work.

But we have another expression—the "Totality of the Symptoms." *Hahnemann, Organon*, Sec. 6, says: "The ensemble or totality of these available signs or symptoms represent, in its full extent, the disease itself—that is, they constitute the true and only form of which the mind is capable of conceiving."

From one point of view the totality is simply the whole number of the symptoms. From another point it is the combination of the symptom elements of a case into a harmonious and consistent whole, having form, coherency, intelligibility, and individuality.

The totality is formed by an inductive process, which consists first, of gaining from the patient and recording an accurate expression and description of all the phenomena which characterize his illness; second, of noting the observations of the patient's attendants and friends; and third, of the physician's own observations, duly expressed and recorded. This is to be done in the light of a true conception of the nature of both a single symptom and the totality of the symptoms.

Dr. P. P. Wells, of ever-revered memory, has given this idea clear, full, and philosophical expression in his famous presidential address before the International Hahnemannian Association in 1881, as follows: "Let us see what is contained in the expression, 'totality of symptoms.' As prescribers, it is with these we have our starting point. Until we have these in our possession, we have no concern with the other factors of the problem we are about to solve. A right understanding of this fundamental expression is necessary before we can take the first step in a true homœopathic prescription. 'Totality of Symptoms'—what does it mean here? All the symptoms of the case is it answered? It means this and more. The 'totality' here means not only the sum of the aggregate of the symptoms, but also this other and most important fact of all, in true homœopathic prescribing, *the totality of each individual symptom of the aggregate group. A single symptom is more than a simple fact; it is a compound made up of a fact with its history, its origin, progress, and conditions attached.* If it be a cause of suffering to the patient, then in it are included all the circumstances of its aggravation or amelioration; as to time of its greatest intensity, position, motion, rest; how affected by eating, drinking, or the performance of any bodily function ; how affected, if at all, by different mental emotions ; or by any other cause of increase or relief of suffering. All this is included in the totality of each single symptom, and without all this the prescriber is ignorant of the ultimate nature of symptom for which he is to find a simillimum."

Symptoms educed in this intelligent and truly scientific manner from patients or provers, form a sound and reliable basis for prescribing. Such a method pursued by those master provers, Hahnemann and Hering, made their provings the model for all successors—a model, alas ! too seldom followed.

Such a method in preparing clinical cases for publication gives us reliable material, in the confirmation of already observed symptoms, as well as new symptoms clinically evoked, worthy of incorporation in our manuals and repertories.

For such work as this, Bönninghausen, Lippe, Wells, Joslin, Dunham, Farrington, and others who have passed on into the larger life, were justly celebrated. They have many able and worthy followers now, and the number is increasing, but the perusal of most of the journals bearing the homœopathic name to-day is distressing to one familiar with the work and methods of those great ones who have been named.

Hahnemann (*Organon*, Sec. 7) calls the totality " this image (or picture) reflecting outwardly the internal essence of the disease—*i. e.*, of the suffering life force." (Fincke's translation.) The word is significant. An image or picture is a work of art, and appeals to our æsthetic sense as well as our intellect. Its elements are form, color, light, shade, tone, harmony, and perspective. As a composition it expresses or represents an idea, it may be of sentiment or fact, but it does this by the harmonious combination of its elements into a whole—a totality. Each element must be given its full value and right relation to the other elements.

Dr. J. H. P. Frost says : " Under the term '*symptoms*' the homœopathician includes all that can be learned respecting the disorder of his individual patient. Hahnemann's minute and exhaustive directions for the examination of a patient amply prove this. These directions comprise not only the totality of the present pathological condition of the patient, *but regard also his past history and that of his parents and grandparents.*"

In our study of *The Organon* we meet another term which requires definition. In Section 153 Hahnemann enjoins us to pay particular and almost exclusive attention to the peculiar, essential, *characteristic* symptoms of a case. The term "characteristic" has come to have a technical meaning. There has been much discussion as to what constitutes a characteristic symptom. Largely upon this point have the existing divisions in our school been made.

We have, first, those who practically ignore Section 153 above quoted, and insist upon what they call the "totality of the symptoms" being made the sole basis of treatment, meaning thereby that every symptom of the case must be covered by the chosen remedy. Theoretically this is true, but much depends upon the definition of the expressions "totality" and "symptoms." Without a true and comprehensive conception of the meaning of these terms, one may gather a mass of indefinite, fragmentary, and unrelated facts, and by following a mechanical method of comparison, degenerate into a mere unintelligent and superficial "symptom coverer."

The second class is composed of those who are known as "keynote prescribers," many of whom, it is to be feared, have no true understanding of the principles underlying the method known as the "keynote system," which originated in the minds of some of the masters of our art, and is based upon Section 153 of *The Organon.* Practically, the majority of such prescribers content themselves with making a superficial examination of a case, seizing upon one or two symptoms which they regard as peculiar, and basing a prescription upon these. The symptoms so selected may or may not be truly characteristic of the case or remedy. The elements upon which a reliable judgment could be based are not present. This is also a perversion of what is essentially a true and valuable method, when rightfully understood.

The third class proudly, and sometimes arrogantly, proclaims itself as "The Physiological School." Pathology is their hobby; recognition by and amalgamation with the allopathic school their ideal.

They also base their prescriptions upon "characteristic symptoms." But to them a characteristic symptom is a general symptom—one common to many patients or many provers. In a proving, the more provers a given symptom is repeated in, the more valuable and characteristic is that symptom. They would exclude from the materia medica all symptoms not experienced by more than one prover, and all symptoms for which a pathological basis cannot be found. In a patient, the symptoms common to the pathological condition—in other words, the diagnostic symptoms—are regarded as characteristic, and made the basis of the prescription, which is very likely to be a massive dose of a crude drug, or a very low dilution, if not a formal prescription to be filled by the nearest druggist, while adjuvants derived from allopathic sources are freely used.

We might make a fourth class of those who are consistent enough, clear-minded, well informed, and *broad* enough to see and accept, not only the sides or portions of truth held by each of the three preceding classes, but the truth as a whole, in its larger aspect, and who thus stand in that liberty which the possession of the truth always gives.

In discussing characteristic symptoms from this larger and truly homœopathic standpoint, Dr. Lippe wrote as follows: "When medicines are submitted to provings upon the healthy, they develop a variety of symptoms in a variety of provers. *Each prover has his own peculiar, characteristic individuality affected by the medicine in a peculiar manner; other differently constituted individuals experience different, yet similar, peculiar symptoms from the same medicine.* There is a similarity and a difference evident on close comparison. In like manner diseases and all other external influences affect different individualities differently and yet similarly. The physiological school and its followers accept in disease only what is general to all those affected by it; in medicinal provings, in the same manner, they accept only that which has been experienced alike by many. In both cases they simply generalize. The homœopathic school reverses this order. Accepting all the symptoms experienced by the differently-constituted provers, *they consider as peculiarly characteristic the individual symptoms of the patient;* those not generally experienced by others suffering from a similar form of disease."

This is individualizing from a Hahnemannian standpoint. One individualizes diseases, the other patients or cases. One is the traditional method, based upon an arbitrary and artificial classification of the grosser phenomena of disease; the other is the scientific inductive method, based upon the ascertained facts of the particular case in hand, in the light of a universal principle, *similia similibus*, or, in other words, the principle of mutual action, upon which every action in the universe takes place.

Again Dr. Lippe says: "In many cases the characteristic symptoms will consist in the result obtained by deducting all the symptoms generally pertaining to the 'disease' with which the patient suffers from those elicited by a thorough examination of the case." He illustrates this by the following case: "The patient is attacked by cholera. All the characteristic symptoms (of cholera) are present; but in this particular case there is also present an unusual noise in the intestines, as if a fluid were being emptied out of a bottle. The discharges come away with a gush. Of what pathological value these symptoms are we know not. Still they form part of the totality which we must cover. Deducting from the totality of the symptoms those common to the disease, we are in possession of the characteristic symptoms of the patient. We now find these two symptoms are also characteristic of *Jatropha-curcas*, and that this remedy, at the same time, has caused symptoms corresponding with the general pathological condition."

We have, therefore, the curative remedy selected by means of two symptoms of no known pathological value and of seemingly trifling character. Yet these two symptoms were what gave the case its individuality, and unerringly pointed out the remedy which corresponded with the totality of the symptoms, according to the principle of *similia similibus*.

Let it be observed that the recognition of these two symptoms as peculiar, or characteristic, depended, first, upon the presence for study and comparison of all the symptoms of the case, and, second, upon the knowledge of what symptoms were common to such cases. The physician must not be ignorant of the pathology, symptomatology, and diagnosis of disease, but he must give these their true place and value.

Dr. Wells says: "Characteristic symptoms are those which *individualize* both the *disease* and the *drug*. That which distinguishes the individual case of disease to be treated from other members of its class is to find its resemblance among those effects of the drug which distinguish it from other drugs. This is what we mean when we assert that with these the law of cure has chiefly to do. When we say 'like cures like,' this is the 'like' we mean." Dr. W. James Blakely wrote: "By the characteristic symptoms we mean those finer and more delicate shades of drug action which are observed in the proving of every remedy. They are symptoms, for the most part, of an apparently trifling nature; symptoms which the prover recorded because he had experienced them, and not from any knowledge of their value; symptoms he was tempted, no doubt, to omit, so slight and unimportant did they appear to him." They are observed as a result of the closest scrutiny and individualization by the prover or by the physician.

Dr. Raue points out that scarcely one of the characteristic symptoms belongs exclusively to a single remedy, and cautions us not to diagnose a remedy on one symptom only, be it ever so characteristic. "While in some cases it may point directly to the remedy, it cannot do so in every case, as it is not rational to suppose that the whole sphere of action of a remedy, which is often extensive and complex, should find its unerring expression and indication in one symptom. But such characteristics are of great aid in the selection of a remedy, as they define the circle of remedies out of which we must select."

There are, however, a very few complete and perfect symptoms which appear under a single remedy only. Such a symptom Dr. Lippe called a *unicum*. They have mostly been discovered, or completed in clinical experience. One such is found under *Staphisagria:* "Backache, worse lying down, at night, compelling to rise at 1 A. M., and relieved by sitting up;" a symptom partly pathogenetic, partly clinical, but as a whole verified by Dr. Lippe in a remarkable case. Another is found under *Kali-bichromicum:* "The posterior wall of the pharynx is dark red, smooth, glossy, distended, traversed by small, bright red vessels; in the middle, a little to the left, is a small crack from which blood oozes." This also has been verified. Upon such a single symptom a prescription may be based in perfect confidence. But they are seldom found.

The principles of the "keynote system," already referred to, are set forth in the following synopsis of an essay by Dr. H. N. Guernsey, the originator of that phrase:

"The term keynote is merely suggestive as used in this connection, the reference being to the analogy between the homœopathic healing art and music. This analogy is shown by the use of other musical terms in medicine, as when a patient speaks of being 'out of tune,' or the physician speaks 'of the tone' of the organism. Disease is defined as a loss of *harmony* in function and sensation. The keynote in music is defined as being 'the fundamental note or tone to which the whole piece is accommodated.' In pathology the term 'pathognomonic symptom' often expresses what might be called the keynote of a disease, or that which differentiates it from other diseases of a similar character. In comparing the symptoms of medicines, we find that each medicine presents, besides the fundamental similarity to all the others, *peculiar differences* from all the others. *These points of peculiar differences are the keynotes to the comparison of such remedies.* The keynote system does not conflict with the doctrine of the necessity for the totality of the symptoms. It is claimed, not that the keynote of the case is to be alone met by the keynote of the remedy, nor that the whole case is to be met by the keynote alone, but simply that the predominant symptom or condition of the case that individualizes it and constitutes its keynote, suggests to the mind a medicine having a corresponding predominant symptom, condition, or keynote, and that, if no error has been made, the totality will be found in the materia medica under that medicine. It is not the totality that directs the attention to a certain remedy. It is always something peculiar in the case, some prominent feature or marked symptom that directs to a certain drug, and the totality afterwards confirms or disapproves the choice. As in the hands of a Leidy or an Agassiz a few bones or teeth, or the scale of a fish are sufficient to unfold a whole chapter in natural history, so in homœopathic practice, by the characteristic keynote emphasized by the patient, the practitioner is enabled to individualize his case, and draw to his aid thus revealed, the corresponding similar remedy having the totality of the case."

Thus we see that by the term "keynote" Dr. Guernsey meant exactly what Hahnemann, Hering, Lippe, and Wells meant by "characteristics;" no more, no less. By the happy choice of a new, striking, and suggestive *name*, Dr. Guernsey exemplified the truth and value of the doctrine referred to, for the widest attention was thereby attracted to the doctrine and to him, and it made him famous. The name he chose proved to be the "keynote" of his own great popularity as an author, a teacher, and a practitioner. A maker of soaps, striking so apt a name for his wares, would have copyrighted it, published it broadcast over the world, and reaped a million dollars as the reward of his sagacity; but his fame would not have been as wide, as secure, and as lasting as that of Dr. Guernsey.

Hahnemann's teaching in this matter has been widely misunderstood. Section 18, in which he declares that "the sum of all the symptoms of each individual case must be the sole indication, the sole guide, in the selection of the remedy," has been regarded as a contradiction of Section 153, where he says that the peculiar, characteristic symptoms "must be almost solely kept in view."

On this point Dr. S. A. Kimball acutely remarks, "In stating that the totality of the symptoms must be the sole indication to direct us in the choice of a remedy, Hahnemann undoubtedly intended to emphasize the idea that we are not to give weight to hypothesis in diagnosis, or to indulge in any theoretical speculations in regard to the case, but to confine ourselves solely to the subjective and objective facts presented to us by the patient. In this way the totality of the symptoms is to be the only *source* of our indications for the selection of the remedy."

A symptom not particularly characteristic in itself may be very peculiar and characteristic in its relation to the other symptoms of the special case under consideration, as in Dr. Lippe's illustrative case, already quoted. This can be determined only by reference to the complete record of the symptoms of the case, known technically as the "totality." All the symptoms must have been gathered and carefully defined before we can proceed with the analysis and comparison by which the characteristics, or keynotes, can be chosen and given their true rank and value. The idea held by many that they can be selected without such an examination is a pernicious one, and it has done much to bring about the deterioration of homœopathic methods and practice which we witness to-day.

Characteristic *specific*, or individual symptoms, must be clearly distinguished from characteristic *generic*, or diagnostic symptoms. The latter have their place and value, but not as a basis for homœopathic prescribing. They are useful negatively, by exclusion, and for purposes of classification. The characteristic symptoms by which we diagnose the *remedy* are entirely different from the characteristic symptoms by which we diagnose the *disease*.

The principles upon which this paper is based may be briefly summarized as follows:

1. Homœopathy is an inductive science, dealing primarily with the facts of experience in sickness, which are called symptoms.

2. The ideal of Homœopathy is healing the sick by the application, in medicine, of the universal principle of Mutual Action, stated by Newton in the formula "action and reaction are equal and contrary," and by Hahnemann in the phrase *similia similibus curantur*.

3. Its basis of action is the totality of the symptoms, stated to be not only the whole number of symptoms in a given case, but the totality of each individual symptom.

4. The perfect remedial correspondent or curative, under the law of similars, technically called the simillimum, is that medicine which has the power of producing in a healthy person symptoms which, in their totality, are most similar, or equal, to those of the patient.

5. The choice of the remedy is made by comparison of the symptoms of the patient with the symptoms of the remedy. This process is facilitated by selecting from the totality of symptoms of the patient characteristics, or keynotes, which are the peculiar symptoms that give the case its individuality and differentiate it from other cases of a similar character, and searching for a medicine having similar characteristics. Such selection of keynote and remedy is afterward verified by comparison of totalities.

6. A characteristic symptom may be defined as one which has been given character and individuality by being precisely defined and set in proper relation to its concomitant symptoms.

